# High Resolution Nonlinear Laser Spectroscopy

**DOI:** 10.6028/jres.093.110

**Published:** 1988-06-01

**Authors:** John C. Wright

**Affiliations:** Department of Chemistry, University of Wisconsin, Madison, WI 53706

Ultra-trace analysis is often limited by the selectivity of an analysis procedure because at low concentration levels, there are a very large number of potential interferences. In spectrochemical analysis procedures, the selectivity is limited by two factors:

1. Spectral congestion where each individual component has a number of transitions and a multi-component sample will contain the contributions from all transitions of all components.
2. Line overlap where each transition has a linewidth that can be greater than the average separation between components in the mixture.

High resolution laser techniques were developed that address the problems listed above. The techniques are collectively called site selective laser spectroscopy. They relied upon the idea that a narrow band laser could be tuned to excite selectively an absorption line of a specific component or site within a sample so the resulting fluorescence spectrum came only from the site or component excited. One could simplify spectral congestion with this approach. One could also eliminate broadening that was caused by inhomogeneities in the samples because excitation within a broadened line would only excite components that had energy states resonant with the laser so the resulting fluorescence spectrum would not reflect the inhomogeneities. These methods were applied to matrix isolation, low temperature organic glasses, Shpol’skii systems, inorganic analysis using precipitates, and supersonic jet spectroscopy to measure a variety of inorganic and organic materials at ultratrace levels. The methods all relied upon sample fluorescence and they failed if the samples were non-fluorescent.

We have recently shown that there is a new family of high resolution laser spectroscopies that have the same capabilities but do not require a fluorescent sample. These spectroscopies are based upon nonlinear mixing where several tunable lasers are focused into a material and new frequencies are formed at all of the sums and differences of the original laser frequencies. This nonlinear mixing is resonantly enhanced when some of the laser combinations match resonances of components in the sample. The nonlinear mixing can be used to perform atomic spectroscopy and molecular spectroscopy. We will concentrate on molecular spectroscopy in this discussion.

One can perform component selection by tuning the lasers to match resonances on one specific component in the sample. One would then expect to have that component contribute dominantly to the nonlinear mixing. One can also eliminate inhomogeneous broadening by tuning the lasers to match the resonances of specific sites within the inhomogeneously broadened line. Again, one would expect that those sites would contribute dominantly to the mixing and the nonresonant sites would be discriminated against.

We have tested these ideas in several model systems using four wave mixing spectroscopy. The two model systems are pentacene doped into p-terphenyl crystals and pentacene doped into benzoic acid crystals where p-terphenyl was added in small amounts to introduce controlled amounts of inhomogeneous broadening. The experiments were done at 2 K to eliminate thermal effects. There are four schemes that one can use to establish resonances with the pentacene molecules. In all of them, one establishes resonances with the vibrational levels, the excited electronic states, and the vibrational levels of the excited electronic state (which we will call vibronic states). The four schemes differ in which states are involved in the resonance associated with the emitted light. If the emitted light involves transitions between two levels that are not initially populated, the technique is classified as a nonparametric process. If one of the levels were initially populated, the technique is a parametric process. Theories for the ideas predict that each possibility will have a different ability to provide selectivity in the measurement.

The pentacene in p-terphenyl system was studied first. Pentacene has four different crystallographic sites in this crystal, some of which differ only slightly from each other. Conventional spectroscopy shows that the transitions from each component appear together in the spectrum and lead to congestion. The nonlinear, four-wave mixing spectroscopy was applied to see if one could produce spectra from the individual sites without contributions from the other sites. We found that one could obtain single site spectra for the vibrational, electronic and vibronic states separately with high selectivity. There were, in fact, no contributions visible in the spectra from even the nearby sites.

The pentacene in benzoic acid doped with p-terphenyl system was studied as a model to see if one could eliminate inhomogeneous broadening. We used both parametric and nonparametric techniques to test the characteristic of each. We found that both methods could eliminate inhomogeneous broadening to the limit of our laser resolution.

This result was a surprise because the theory for the method predicts that only nonparametric methods could successfully eliminate inhomogeneous broadening. The explanation for the observation of parametric nonlinear mixing removing inhomogeneous broadening could only rest on the possibility that the character of the mixing changes as a function of the laser power because of the participation of excited state populations. A new theory was developed that treated saturation effects correctly and described excited state populations. The theory predicts intensity dependent changes in the relative intensity of the narrowed line to the non-narrowed line. It also predicts that the relative intensities are dependent upon the amount of inhomogeneous broadening. These predictions are consistent with the experimental observations.

At this point, we have succeeded in developing a new class of high resolution laser spectroscopy methods based on nonlinear mixing. The methods do not require that the sample emit light. We have applied the methods to molecular and atomic spectroscopy and are now ready to extend the methods to realistic samples and mixtures. The methods are capable of measuring both bulk concentrations and surface concentrations. Surface specific analysis is possible because nonlinear mixing processes that are based on three wave mixing are only allowed for noncentrosymmetric systems. Thus, three wave mixing will not be observed for the bulk of a material but it will be observed at the interfaces between materials. It is also important to realize that the selectivity is determined on the time scale of the lasers and is not limited by the lifetime of excited states. An environment can change while the excited state is occupied and the changes will act to broaden the spectral line. However, if a picosecond excitation is used in the line narrowing experiment, the motion will be frozen and one can eliminate the broadening. This idea has important implications for solution spectroscopy because the motion within a solvent acts to broaden all of the lines that one would normally observe. There is, in fact, a rich field of opportunity for the development of these spectroscopies that is only being hinted at in our present work and it will be exciting to see what capabilities lie in the future.

